# Cholera

**Published:** 1872-10

**Authors:** W. S. Ely

**Affiliations:** Rochester, N. Y.


### Selections.

*Cholera.* By W. S. Ely, M.D., Rochester, N. Y. (Read before the Medical Association of Central New York.)

Gentlemen—As the last named member of your "Committee on Cholera," I beg to state that I assumed that the gentlemen preceding me on the committee, would furnish you exhaustive reports. I have, therefore, endeavored only to give in brief the conclusions reached from reference to the most recent and trustworthy published views upon — First, The origin and nature of Cholera; Second, Its propagation; Third, Its salient pathology; Fourth, Its treatment; Fifth, Its prevention.

FIRST—ITS ORIGIN AND NATURE.

Cholera is known to have existed epidemically and endemically in India for hundreds of years. Though generally supposed to have first appeared in Europe and America in the present century, there are those who believe it prevailed in Europe two or three hundred years ago, while a few medical historians think that the disease was known to Hippocrates. All are familiar with its history in the past fifty years, which embraces its progress from the far East, westward, and the invasion of this country in 1831 and 1832—its subsequent disappearance—to reappear in 1849, 1850, 1851 and 1852—and again in 1866.

With regard to the nature of cholera, speculation is abundant and facts are few. Though nearly all investigators believe in a specific poison, which is (material) organized—all potent—its essence has not yet been grasped. The *germ* theory has intelligent propounders, and may be said to be in favor. Professor Hallier, of Jena, is its most strenuous advocate. Having found in cholera stools certain microzymes, he builds his castle thus: Rice grains steeped in cholera discharge germinate, but the plants are sickly, and become covered with one of the fungi known as "smuts." Cholera originates on the banks of the Ganges, where the rice plant abounds. The contagion of cholera is developed in a peculiar fungus, parasitic on the new plant, and the same material which, brought into contact with germinating grain, produces a smut, when introduced into the intestines, generates cholera. This beautiful discovery seems not to have been corroborated by Hallier's co-workers.

Aitken is afraid to commit himself, and simply says: Cholera is a disease of miasmatic orgin—probably now indigenous in Great Britain. Neimeyer don't like to experiment with the fertilization of rice with cholera discharges, and so says he won't dwell on Hallier's views, as he is not competent to decide them; but he does not hesitate to add: "Cholera, with us, is never miasmatic or indigenous, but is always due to an exotic parasite, brought us by cholera patients, and finding, for a time, a suitable soil and favorable circumstances for increasing." Pettenkoffer, in his latest work, affirms that cholera has its origin in a specific infective substance, which certain regions in India have produced for centuries. As it does not develop to the same extent every year, nor in every region, dying out and reappearing, you must assume not only the existence of a specific germ, or infective substance, but of an actual determinative local and periodical substratum, without which the specific cholera germ cannot produce cholera in the human subject. Neither the germ alone nor the local and seasonal conditions can produce the disease. It is only the product of the action of one on the other. We may accept it as a scientific probability that the cholera germ, the local and seasonal substratum, and the real cholera poison, the resultant, are of organic origin.

SECOND—THE PROPAGATION OF CHOLERA

includes the question of its contagious or infectious properties—about which there has been much dispute, due to misuse of the terms *contagion* and *infection*. These words have no clearly defined signification, often being employed synonymously. It is admitted that cholera is not contagious—in the sense of being communicated immediately from the sick to the well. Fresh cholera discharges have been fed to animals, and swallowed by men, without injury; those caring for the sick are not especially affected; the dissection of the dead is not dangerous. In short, there is no immediate contagion. How then is the disease propagated? An easy question to put—a difficult one to answer. Earth, air, water, fomites, human intercourse, have separately and combined been implicated—and I am of the opinion that all may be concerned in its transmission. The learned Graves very early said that "cholera seemed regulated by no common physical circumstance except human traffic and human intercourse." Hours might be spent in elaborating this statement, by tracing epidemics along great routes of travel, their progress marked by the decimation of cities, pilgrims, armies. Our observation is as constant as it is significant, viz: that the disease has been most destructive when there has been the greatest disregard of hygienic laws. We are forced to believe, provisionally, that cholera is propagated by indirect contagion—through the medium of the discharges—conveyed either through drinking water (Snow's theory, exemplified in London in 1849, and again in Brooklyn in 1866), or through dry discharges blown about, or through exhalations from earth saturated with discharges. (Pettenkoffer's view, with abundant observation to confirm it). Mr. Simon, of England, reviewing the epidemic of 1866, says: "It cannot be too distinctly understood that the person who contracts cholera in this country, is demonstrated, with almost absolute certainty, to have been exposed to excremental pollution. That which gave him cholera was, mediately or immediately, cholera-contagion discharged from another's bowels; that the causes of cholera in England are excrement-sodden earth, excrement-reeking air, excrement-tainted water," to which may be added excrement-soiled clothing, bedding and utensils of the sick. Neimeyer plants himself strongly on this doctrine. In a small epidemic at Griesswald, the investigation of every case showed that the patient had used a privy of some house containing cholera patients, or indirectly communicating with it. He affirms that a person having simple choleraic diarrhœa may infect a district through which he may be passing. Abundant proof of the value of this theory of propagation, is found in the efficiency of preventive measures indicated by it. C. McNamara, of Calcutta, in a large treatise of 527 pages, issued in 1869, based on extensive study of cholera in India, says that "every outbreak of the disease beyond the confines of British India, may be traced back to Hindostan through a continuous chain of human beings similarly affected, or through contaminated water or fomites." He entirely ignores all other causes—never finding the disease to spread more rapidly than can be explained by this view. The New York Metropolitan Health Board, in 1866, acted most successfully upon the conclusion which it deemed previous epidemics had established, viz: "that the discharge of cholera patients impregnating soil, water, privies, sewers, constitute the positive, and as far as now known, the only means of propagating Asiatic cholera."

THIRD—WHAT ITS SALIENT PATHOLOGY.

The poison which produces the profound disturbance in cholera has been supposed to act primarily upon the blood as a rapid disorganizer, or primarily upon the nervous system, derangement of which excites blood changes. A few hold that we have to deal with a local intestinal disease. (Snow's theory). Everything points to a specific poison of intense energy, which rapidly takes from the blood its water and salts, early affects the kidneys, causing suspension of urinary secretion, and as the algid state is developed, lowers the temperature. I purposely omit symptoms, and pause not to detail frequently observed appearances of intestines, liver and kidneys, but desire to point especially to the pulmonary circulation, with reference to a theory of collapse now largely accepted. Collapse is not chiefly due to the drain of liquid from the bowels, as supposed, because collapse and discharges are often in inverse ratio. Stimulants do no good, and recovery is at times too rapid to be consistent with this view. What then, is the explanation? It is that the essential cause of cholera collapse is an impeded circulation through the lungs, due to the contraction of minute pulmonary arteries upon poisoned blood. Autopsies of those dying in collapse, show always the left heart empty, while the right heart and systemic veins are distended. There seems to be a "stop-cock" action of minute vessels upon the poisoned current, by which it is arrested before it can reach the capillaries. A scanty oxygen-bearing stream results, and consequent lowered temperature, with suppression of the secretions dependent on oxidation, bile and urine. It is this pathological observation of the pulmonary condition that gives the strongest coloring to Johnson's "eliminating treatment." The discharges from the stomach and bowels being the means by which the poison is thrown off, we must aid them; but, alas! the poor patient can neither withstand the poison nor the shock to his system which its expulsion imposes.

### FOURTH—THE TREATMENT OF CHOLERA.

With diverse views as to the causation and pathology of the disease, we expect a corresponding difference in methods of treatment. As in well established cases about as many have died under one plan of management as another, not much encouragement can be offered for any particular line of practice. If you wish to give calomel in doses of twenty grains or one-fourth grain, if you make opium or ipecac your sheet anchor, or from a malarial stand point give quinine, if you rely on castor oil and cold water, after Johnson, or decide to inject saline solutions into the veins for the deceptive improvement it will furnish, or, finally, if you fold your hands and employ no medicine, I will furnish you respectable authority for any of these plans. This is not encouraging, and should stimulate our efforts to improve our therapeutical resources. The judicious treatment of symptomatic indications will save lives, and in the absence of more definite knowledge, should be our rule.

### FIFTH—ITS PREVENTION.

Sad, indeed, gentlemen, would be the whole history of cholera did not great encouragement come to us under this head. Though study and research have confessedly added nothing to our ability to cope with the fully formed disease, they have resulted in incalculable benefit by pointing out the practicability of its prevention. The arrest of the premonitory diarrhœa is, in the large proportion of cases, possible, and in every cholera epidemic the public should be instructed in reference to it, by published statements or house-to-house visitation. Remember that Fary, the great English statician, believes that "the evidence of cases of algid cholera without premonitory diarrhœa is imperfectly established." A cholera scourge is always preceded and accompanied by an increase of intestinal disorders, and these are produced and aggravated by unsanitary conditions. It is now established beyond question that crowding of population, filthy streets, decomposing organic matter, and, above all, foul privies and defective or clogged sewers, when taken with high temperature and moist air, are productive of diarrhœal disorders, and, in cholera years, favor the rapid spread of the epidemic. New York City has its "diarrhœal fields," as they are called, where the above conditions prevail every year, occasioning great mortality. Witness the cholera epidemic of 1866. It invaded these districts nearly exclusively, and was only controlled by a thorough police and lavish use of disinfectants. It is not my aim to cite proof for these assertions; it is furnished in abundance in recent reports on cholera epidemics. Let one instance suffice. In the 1866 epidemic on Blackwell's Island, there were 123 deaths in nine days. On the fourth day, when the epidemic was at its height, Dr. F. H. Hamilton gave his pledge to the Board of Commissioners that he would drive the cholera from the workhouse in from three to five days, if certain instructions were carried out. On the fifth day the pledge was redeemed and not another case occurred. Turning all the inmates out of doors, and disinfecting every stool, every latrine, every vessel, accomplished this result. Of the epidemic in New York City, it was said that "events proved that vigilance was the price of safety. If any one doubts the necessity and usefulness of thorough cleansing and disinfection, the experience of September would remove such doubts." The problem of disinfection, then, is the one we should address ourselves to in anticipation of cholera, and in its actual presence. Thorough use of sulphate of iron, the chlorine and bromine compounds, carbolic acid, and coal tar agents, and fumigation of houses, conjoined with organized inspection, will rob the epidemic of its terror and its fatality. Quarantine should be enforced, but not regarded as an absolute safeguard.

In the foregoing brief abstract of views, no complete discussion of any branch of the subject has been attempted, and reference to diagnosis, symptomatology and sequelæ has been purposely omitted, because upon these heads text-books are in full accord.

